# The role of retinoic acid receptor-related orphan receptors in skeletal diseases

**DOI:** 10.3389/fendo.2023.1302736

**Published:** 2023-11-08

**Authors:** Yifan Zhang, Jun Ma, Xingfu Bao, Min Hu, Xiaoxi Wei

**Affiliations:** ^1^ Department of Orthodontics, Hospital of Stomatology Jilin University, Changchun, Jilin, China; ^2^ Department of Oral Anatomy and Physiology, Hospital of Stomatology Jilin University, Changchun, Jilin, China

**Keywords:** RORs, bone homeostasis, osteoporosis, rheumatoid arthritis, osteoarthritis

## Abstract

Bone homeostasis, depending on the balance between bone formation and bone resorption, is responsible for maintaining the proper structure and function of the skeletal system. As an important group of transcription factors, retinoic acid receptor-related orphan receptors (RORs) have been reported to play important roles in bone homeostasis by regulating the transcription of target genes in skeletal cells. On the other hand, the dysregulation of RORs often leads to various skeletal diseases such as osteoporosis, rheumatoid arthritis (RA), and osteoarthritis (OA). Herein, we summarized the roles and mechanisms of RORs in skeletal diseases, aiming to provide evidence for potential therapeutic strategies.

## 1 Introduction

Bone homeostasis is an essential dynamic process of bone modeling and remodeling (1), which requires accurate regulation by a variety of transcription factors as well as signaling pathways (2, 3). Retinoic acid receptor-related orphan receptors (RORs) are ligand-dependent transcription factors and can regulate osteogenesis and osteoclastogenesis in bone metabolic disorders such as osteoporosis (4). In addition, the RORs, particularly RORγ, mediate the inflammatory response and lead to bone destruction in inflammatory bone diseases such as rheumatoid arthritis (RA) and osteoarthritis (OA) (5). In this review, we discussed the effect of RORs on maintaining bone homeostasis and summarized prospective therapeutic strategies targeting RORs for common bone diseases.

## 2 The RORs

**Figure 1:**
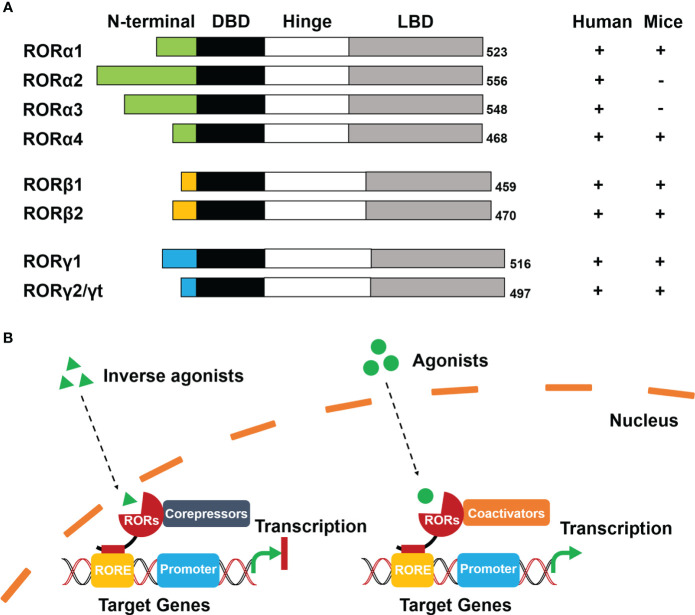
Schematic diagram of structure and ligand-dependent mechanism of RORs. **(A)** Structure: Four functional domains constitute RORs, including a distinct N-terminal domain, a highly conserved DNA-binding domain (DBD), a ligand-binding domain (LBD), and a hinge domain. The numbers at the tail represent the total number of encoded amino acids in the corresponding ROR isoforms. **(B)** Mechanism: Inverse agonists trans-activate RORs to recruit co-repressors and inhibit transcription of target genes (left). Agonists activate RORs to recruit co-activators and initiate transcription of target genes (right).

RORs are a subgroup of the thyroid hormone receptor and belong to the orphan nuclear receptors (NRs) (6). The RORs contain three members: RORα (same as RORA, NR1F1), RORβ (same as RORB, NR1F2), and RORγ (same as RORC, NR1F3). It has been reported that each member has several isoforms that differ only at their N-terminus, and these isoforms are expressed in different mammalian species. Specifically, RORα contains RORα1–4 in humans, but only RORα1 and RORα4 are expressed in mice. Both RORβ and RORγ generate two isoforms (RORβ1/RORβ2 and RORγ1/RORγ2, RORγ2 is also known as RORγt) in humans and mice (7) (**Figure 1A**).

The modular structure of RORs is typical of NRs and consists of four functional domains: a distinctive N-terminal domain, a highly conserved DNA-binding domain (DBD), a ligand-binding domain (LBD), and a hinge domain (**Figure 1A**). Differences in sequence of the N-terminal domain determine the isoforms of RORs. ROR response elements (ROREs) have an AGGTCA consensus motif with an A/T-rich region. With two highly conserved zinc finger motifs, The DBD is responsible for recognizing the AGGTCA consensus region in ROREs as a monomer. The LBD is the most important functional domain that is crucial for ligand-binding and facilitates the recruitment of cofactors in transcriptional regulation. The hinge domain linking DBD with LBD stabilizes the RORs’ protein structure (8).

Even though evidence has indicated that RORs can be self-activated without any ligands or other potential stimulators, RORs transcriptional regulation is primarily ligand-dependent (6, 7). Cholesterol and its metabolites were firstly identified as natural ligands by crystallography studies (8). Melatonin was once considered as an endogenous ligand of RORβ. Although it could bind to RORβ directly, the result was not reproducible and therefore has been withdrawn (9). Apart from natural ligands, synthetic ligands have made significant progress (10). According to conformational changes of RORs, the ligands include agonists, antagonists, and inverse agonists (11).

RORs interact with co-repressors as well as co-activators and function as repressors or activators of gene transcription respectively. When inverse agonists enter the nucleus, RORs can be trans-activated and recruit co-repressors, such as the nuclear receptor co-repressor (NCoR) and the silencing mediator for retinoid and thyroid hormone receptor (SMRT) (4). The co-repressor complexes then combine with the RORE of target genes that subsequently prevent the promoter initiation and suppress the transcription. In contrast, agonists activate RORs with conformational changes resulting in the dissociation of co-repressor complexes and the recruitment of co-activators, such as the p160 steroid receptor co-activator (SRC) family, p300, and BRG2 complex (6, 12). The formation of co-activator complexes eventually facilitates transcriptional regulation by enhancing the promoter of target genes (**Figure 1B**).

The RORs serve functions in several physiological processes such as cell division and differentiation, circadian rhythm, metabolism, and immune regulation (12). ROR family members and their isoforms exhibit a distinct tissue-specific pattern of expression and play different roles in pathological processes. Specifically, RORα is widely distributed in multiple tissues, including the liver, skeletal muscle, skin, and adipose, which is mainly associated with circadian rhythm abnormalities, tumorigenesis, and metabolic diseases. In addition, RORα is highly expressed in the pre-hypertrophic and hypertrophic chondrocytes of the growth plate, suggesting that RORα may be a regulator of hypertrophic differentiation of chondrocytes and contribute to endochondral ossification (13, 14). RORβ is expressed restrictedly in the brain, retina, bone, and pineal gland, and is essential for retinal cell survival and circadian rhythm regulation (4). Moreover, the two isoforms of RORγ differ in the expression with distinct N-terminus. RORγ1 is expressed in various tissues such as skeletal muscle tissue, liver, and kidney, while RORγ2 (same as RORγt) is exclusively expressed in immune cells and plays prominent roles in thymocyte development (12).

## 3 Role of RORs in skeletal diseases

Skeletal diseases, such as osteoporosis, rheumatoid arthritis, and osteoarthritis, significantly impair individuals’ health and quality of life. Patients may suffer from long-term pain, limitation of movement, and hypofunction. Studies have found that RORs play an important role in bone metabolism and maintenance of bone homeostasis. Interestingly, ROR family members play different roles in regulating osteogenesis. Most studies believe that RORα mainly promotes osteogenesis. On the contrary, RORβ inhibits osteogenic differentiation. RORγ regulates bone balance by participating in the inflammatory response and stimulating osteoclastic differentiation. Herein, we summarize the role of RORs in common skeletal diseases.

### 3.1 Osteoporosis

Osteoporosis is one of the most common bone metabolic disorders, characterized by bone loss and microstructural changes, which in turn increase the risk of fractures (15, 16). Osteoporosis occurs when the rate of bone resorption consistently exceeds the rate of bone formation. RORs affect the occurrence and progression of osteoporosis by regulating the proliferation and differentiation of skeletal cells and multiple osteogenic signaling pathways.

#### 3.1.1 RORα in osteoporosis

Studies have shown that RORα positively regulates osteogenic differentiation *in vitro* and *in vivo*. Spontaneous mutations of the *Rorα* gene were identified in a staggered (sg) mouse strain. Homozygous staggerer (sg/sg) mice showed limited skeletal development with a reduction in long bone thickness and bone mineral density (17, 18). Application of cholesterol sulfate, a RORα agonist, prevented bone mass loss in the inflammation-mediated or ovariectomy (OVX) -mediated osteoporosis models (19). Studies in goats have also confirmed a substantial association between RORα and growth traits including height and bone length (20). *In vitro* evidence suggested that RORα was highly expressed in human mesenchymal stem cells (hMSCs) and that the expression level increased with osteogenic differentiation (17, 21).

**Figure 2:**
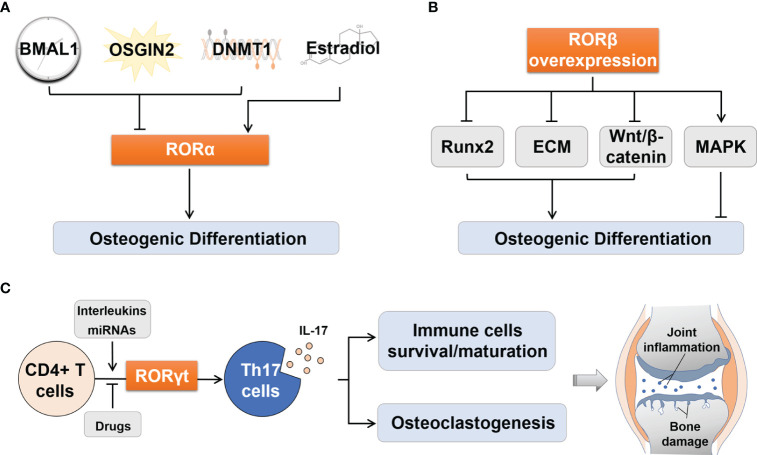
Regulation of osteogenic differentiation by RORα and RORβ and of Th17 cell differentiation by RORγt. **(A)** Upstream effectors including BMAL1, OSGIN2 and DNMT1 could suppressed osteogenic differentiation by inhibiting RORα, while Estradiol activated RORα to promote osteogenic differentiation. **(B)** RORβ overexpression inhibited osteogenic differentiation by inhibiting Runx2 transcription, disrupting ECM synthesis, inhibiting Wnt/β-catenin pathway, and promoting proliferation through the MAPK pathway. **(C)** RORγt regulates the process of differentiation from CD4+ T cells to Th17 cells, and the consequent IL-17 secretion. The latter can influence the survival and maturation of immune cells and osteoclastogenesis, which thus aggravates joint synovial inflammation and bone damage in RA patients. BMAL1, Brain and muscle ARNT-like protein 1; DNMT1, DNA methyltransferase 1; Runx2, Runt-related transcription factor 2; ECM, Extracellular matrix; MAPK, Mitogen-activated protein kinase; Th17, T helper 17; IL-17, Interleukin 17; miRNAs, MicroRNAs.

Several upstream signaling pathways are mediated by RORα in osteogenic differentiation (**Figure 2A**). (1) Circadian clock system: Brain and muscle ARNT-like protein 1 (*Bmal1*) knockout mice exhibited osteoporosis (22). Further studies found that BMAL1 blocked the Wnt/β-catenin signaling pathway by inhibiting the expression of RORα, thereby preventing the osteogenic differentiation of bone marrow mesenchymal stem cells (BMSCs) (23). (2) Oxidative stress: It has been reported that oxidative stress is a key trigger of osteoporosis. OSGIN2, an inducer of oxidative stress, prevented osteogenic differentiation of BMSCs by inhibiting RORα expression (24, 25). (3) DNA methylation: Histone methylation has been recognized as an important modulator in the osteogenic differentiation of MSCs (26). RORα mediated DNA methyltransferase 1 (DNMT1) to control the DNA methylation of MSCs, thereby suppressing chondrogenic and osteogenic differentiation (27). (4) Estrogen: Estrogen plays a significant role in regulating bone metabolism (28). Estradiol is one of the Estrogen’s natural products, which could activate RORα and promote osteoblast differentiation (29).

There are several main mechanisms underlying the downstream pathway of RORα. One is directly promoting the transcription of osteogenic differentiation marker genes, such as alkaline phosphatase (*ALP*), collagen type I *(COL I*), bone sialoprotein (*Bsp*), and dentin matrix protein 1 (*DMP1*) (17, 21, 30). The second is activating osteogenic differentiation signaling pathways, such as Wnt/β-catenin and BMP2/Smad1/5/9 pathways (17, 29). Furthermore, RORα can associate with a variety of cell types and influence the microenvironment directly or indirectly. For instance, in the diabetic bone defect model, underexpression of RORα in macrophages inhibited the transcription of serum C-C motif chemokine 3 (*Ccl3*) and interleukin-6 (*Il-6*), which led to the inability of macrophages to convey migration and aggregation signals to BMSCs, ultimately leading to diabetic bone aplasia (31).

However, it has also been reported that RORα negatively regulates osteogenic differentiation. For example, transient overexpression of RORα suppressed osteocalcin (*Ocn)* transcription induced by vitamin D3 and consequently inhibited osteogenesis (17). In addition, bisphenol A (BPA), a ROR agonist, could promote bone resorption (32). The controversial results may be due to the time and extent of RORα expression in two different ways of both bone formation and resorption in bone metabolism.

#### 3.1.2 RORβ in osteoporosis

In contrast to RORα, RORβ negatively regulates osteogenic differentiation and promotes the progression of postmenopausal or age-related osteoporosis. Deletion of the *Rorβ* gene could prevent bone loss in aged mice (33). RORβ expression was significantly increased with age in the BMSCs from postmenopausal women, which was consistent with the mouse models of age-related osteoporosis (34, 35). Data on overexpression or knockdown of RORβ in osteoblastic MC3T3-E1 cells also suggested its negative effect on osteogenic differentiation (33, 36).

The following mechanisms may explain the negative effects of RORβ on osteogenic differentiation (**Figure 2B**). (1) Runt-related transcription factor 2 (Runx2) is an essential transcription factor that drives the phenotypic expression of osteoblasts. Its absence can lead to a complete loss of ossifying capacity (37). When the expression of RORβ was up-regulated in MC3T3-E1 cells, transcription of *Runx2* and its target genes *Ocn* and osterix (*Osx*) were significantly inhibited (34). (2) Transforming growth factor β (TGF-β) and bone morphogenetic protein (BMP) control the formation of extracellular matrix (ECM) and provide a framework for bone mineral deposition (38). Studies have shown that overexpression of RORβ upregulated the expression of these cytokines and then interfered with ECM synthesis, thereby inhibiting bone mineralization (36). (3) RORβ down-regulated the Wnt/β-catenin signaling pathway by inhibiting the activities of Wnt downstream target genes transcription factor 7 (*Tcf7*) and osteoprotegerin (*Opg*), which disrupted the balance between bone formation and bone resorption, leading to bone loss (33). (4) RORβ could stimulate osteoblast proliferation through the mitogen-activated protein kinase (MAPK) pathway and prevent osteoblasts from exiting the cell cycle, thereby delaying osteogenic differentiation (36). In addition, RORβ has been identified as a molecular target of miR-219a-5p in recent years, which is involved in the development of osteoporosis (39).

#### 3.1.3 RORγ in osteoporosis

Most studies on the effect of RORγ on osteoporosis were limited to RORγ2 (RORγt). The main mechanism is to act as a specific transcription factor of immune cells, especially T helper cell 17 (Th17), and mediate the differentiation of Th17 cells to secret interleukin 17 (IL-17), thereby enhancing inflammatory response (5). In recent years, it has been found that T cell-mediated inflammation plays an important role in the progression of osteoporosis (40, 41). In human and mouse models, RORγt affected bone loss in postmenopausal osteoporosis caused by estrogen deficiency (42, 43). The production of IL-17, mediated by RORγt, directly stimulated osteoclastogenesis by upregulating the expression of RANKL (44, 45). IL-27 inhibited the differentiation of Th17 cells in OVX mice by inhibiting the transcription factor RORγt (46). In addition, as one of the areas of scientific interest in recent years, intestinal flora plays a key role in regulating bone homeostasis through the gut-bone axis (47). The treatment of Lactobacillus rhamnosus GG (LGG) stimulated Th17 cell differentiation with the alteration of RORγt expression, and had an advantage in osteogenic promotion, which may account for the amelioration of osteoporosis in OVX model rats (48, 49). Another popular probiotic, Bifidobacterium longum (BL), appeared its immunomodulatory potential to the Th17 cell differentiation that suppressed osteoclastogenesis in postmenopausal osteoporosis (50).

### 3.2 Rheumatoid arthritis

Rheumatoid arthritis is a systemic autoimmune disease characterized by initial synovial inflammation followed by cartilage degeneration and subchondral bone destruction (51, 52). The promotion of osteoclast formation and differentiation is the main reason for bone erosion (53). Immune cells, especially Th17 cells, are activated and concentrated in the inflamed synovium, contributing to persistent joint inflammation and bone erosion in RA (54). Among RORs, many studies indicated the roles of RORα and RORγt in inflammatory diseases like RA and OA, whereas little has been done on RORβ.

#### 3.2.1 RORα in rheumatoid arthritis

RORα is involved in the inflammatory response stage of RA and the regulation of osteoclast activity in bone destruction. RORα is expressed in Th17 cells and the expression is elevated in collagen-induced arthritis (CIA) mice (55). RORα can act as a transcription factor to mediate the differentiation of Th17 cells. The expression of RORα and RORγt together promoted the differentiation of Th17 and significantly up-regulated the expression of IL-17, thereby promoting inflammatory response (56). However, some studies have also reported that RORα is a negative regulator of inflammatory response. Compared with controls, the expression level of RORα in osteoblasts of RA patients was lower (30). The RORα agonist, cholesterol sulfate, could reduce joint inflammation and bone destruction in CIA mice by inhibiting RORγt expression and Th17 cell differentiation (57). Differences in the disease itself and the immune microenvironment determine the cellular response to any stimulus, which may help to partially explain the different mechanisms by which RORα regulates inflammation in Th17 cell differentiation. In addition, RORα is involved in the process of bone resorption in RA. RORα agonists prevented osteoclastogenesis and induced osteoclast apoptosis in CIA mice (57). However, direct depletion of *Rorα* did not affect the agonist-ligand-mediated inhibition of osteoclast differentiation (19). This suggested that RORα agonists might be involved in RA osteoclast survival and differentiation in a RORα-independent manner.

#### 3.2.2 RORγ in rheumatoid arthritis

RORγt, as a lineage-specific transcription factor of Th17 cells, regulates the differentiation of CD4+ T cells into Th17 cells, then promotes the production of IL-17, and finally aggravates joint inflammation and bone destruction in RA (58) (**Figure 2C**). In RA patients, the aggravation of inflammation is closely related to the increase in the proportion of Th17 cells and the expression levels of RORγt and IL-17 (5, 59).

Several upstream signals including cytokines, RA drugs, and microRNAs (miRNAs), can regulate RORγt expression and Th17 cell differentiation. Specifically, pro-inflammatory factors, such as IL-6 (60), IL-23 (61), and tumor necrosis factor-α (TNF-α) (62), transmitted inflammatory signals to Th17 cells. Some natural and synthetic RA drugs, such as digoxin and pioglitazone, reduced the expression levels of RORγt and IL-17, thereby alleviating inflammation and preventing further bone destruction (63–67). miRNAs regulating RORγt expression, such as miRNA-16 (68), miRNA-301a-3p (69), miRNA-34a (70), and miRNA-146a (71, 72), were also involved in RORγt-mediated regulation of Th17 cell differentiation during RA progression. In addition, the RORγt-CCR6-CCL20 axis might be involved in the directional migration of Th17 cells toward synovial inflammation (73). Furthermore, epigenetic modifications can regulate RORγt activity through post-translational mechanisms such as ubiquitination and acetylation (74). During the early stages of RA activity, DNA methylation of RORγt DNA sequences was enhanced (75). TNF-α inhibitors could attenuate the histone acetylation modification on the RORγt promoter and reduce its expression in Th17 cells (62).

In RA, upstream cytokines stimulated signal transduction and activation transcription factor 3 (STAT3) into the nucleus and initiated RORγt activation (61, 65). RORγt and STAT3 could bind in the IL-17 promoter region and thus regulate the production of IL-17. In the downstream pathways, IL-17 overexpression promoted the survival and maturation of fibroblasts and immune cells in the synovium, thereby aggravating synovial inflammation [90], which ran through the whole process of RA (76). At the same time, increased IL-17 levels might promote osteoclast generation through the RANKL/OPG signaling pathway, thereby aggravating subchondral bone erosion (77). Metallothionein-1 (MT-1) inhibited Th17 differentiation by reducing the expression of STAT3 and RORγt, and significantly suppressed synovial inflammatory response and subsequent bone destruction (78).

### 3.3 Osteoarthritis

OA is a chronic degenerative arthritis that affects all joints and is characterized by cartilage degradation, subchondral bone remodeling, and bone mineralization (79). Different from RA, where synovial inflammation is the main pathological change, cartilage degeneration is the pathological center of OA (80). The molecular mechanisms underlying OA pathophysiology are poorly understood. OA was previously considered to be a non-inflammatory joint disease. However, recent studies have identified proinflammatory cytokines and immune inflammatory cells as key mediators of cartilage damage in OA (81). OA and RA share some risk factors and pathogenic features, and they both exhibit highly inflammatory features driven by CD4+ T cells (82). The expression of IL-17 in the synovial tissue of OA and RA patients was increased. This eventually led to increased joint inflammation and bone erosion (83).

It has also been suggested that metabolic disorders may also be an important mechanism leading to this disease. RORα can significantly affect the progression of OA through cartilage-specific cholesterol metabolism. In the CH25H-CYP7B1-RORα axis of cholesterol metabolism, RORα acted as a substrate for cholesterol hydroxylase (CH25H and CYP7B1) (84). High cholesterol and its metabolites could directly activate RORα in chondrocytes, which could bind to the promoter of cartilage matrix catabolic factors and activate its transcription, leading to cartilage damage (85, 86). In addition, RORα accelerated cartilage matrix degradation through the IL-6/STAT3 signaling pathway (87, 88).

## 4 Therapy strategies targeting RORs in skeletal diseases

Therapeutic applications targeting RORs have developed along with the identification of ROR ligands including agonists, inverse agonists, and antagonists. Specifically, ROR agonists enhance the transcription levels of target genes by either promoting the recruitment of coactivator complexes or preventing the recruitment of corepressor complexes, whereas ROR inverse agonists do the opposite. ROR antagonists reduce the transcription by preventing the recruitment of any complexes (11, 89).

Since the first ROR agonist was identified, RORs have been investigated as a potential therapeutic target for various diseases (90, 91). Among bone diseases, inflammatory arthropathy has received the most attention. Treatment with RORα agonist cholesterol sulfate prevented osteoclast osteogenesis in CIA mice and protected against bone loss in postmenopausal osteoporosis (19). A dual inverse agonist of RORβ and RORγt, *N*-(5-(arylcarbonyl)thiazol-2-yl)amides, has been identified to exhibit therapeutic potential in CIA mice (92). Compared with RORα and RORβ, the treatment targeting RORγt is more commonly used in inflammatory and autoimmune arthropathies.

Several natural compounds have been identified as RORγt-specific modulators that can be used in the treatment of autoimmune diseases. Digoxin was the first RORγt inhibitor reported to reduce inflammation in CIA mice (66), however, its disadvantages were obvious cytotoxicity and limited therapeutic index. In addition, ursolic acid had anti-RORγt activity, but it could also activate glucocorticoid receptors as a side effect (93). To solve these problems, the synthesis of selective RORγt small molecule modulators has become a promising treatment for autoimmune diseases such as RA (94). *In vivo* data showed that amide drugs had good clinical efficacy as effective RORγt inhibitors (95). Phenylenediamine derivative of RORγt inhibitor reduced the severity of arthritis symptoms in mice (96). Moreover, imidazolopyridine analogs, a RORγt selective inverse agonist, alleviated the pathological symptoms of adjuvant arthritis (AIA) in rats (97, 98). Recently, three azacyclic inverse RORγt agonists were identified by structure-activity relationship studies, which showed biologically similar efficacy in preclinical models of RA (99).

## 5 Discussion and conclusion

In summary, as transcription factors, RORs directly regulate osteogenic differentiation and osteoclastogenesis and associate with other factors or signals to indirectly mediate the destruction of cartilage and bone by immune-inflammatory responses. In terms of selective molecular modulators, RORγ has made remarkable progress in autoimmune diseases. However, on the pharmacological front, there are concerns about the safety and efficacy of existing small-molecule drugs targeting RORγ. In addition, the effects of administration form and ROR binding mode on clinical efficacy remain to be further studied.

## Author contributions

YZ: Conceptualization, Writing – original draft. JM: Conceptualization, Funding acquisition, Writing – review & editing. XB: Writing – review & editing. MH: Writing – review & editing. XW: Conceptualization, Funding acquisition, Writing – original draft, Writing – review & editing, Supervision.
